# Atrial Natriuretic Peptide Acts as a Neuroprotective Agent in *in Vitro* Models of Parkinson’s Disease via Up-regulation of the Wnt/β-Catenin Pathway

**DOI:** 10.3389/fnagi.2018.00020

**Published:** 2018-02-01

**Authors:** Arianna Colini Baldeschi, Eugenia Pittaluga, Federica Andreola, Simona Rossi, Mauro Cozzolino, Giuseppe Nicotera, Gianluca Sferrazza, Pasquale Pierimarchi, Annalucia Serafino

**Affiliations:** Institute of Translational Pharmacology, National Research Council of Italy, Rome, Italy

**Keywords:** Wnt/β-catenin pathway, neurodegeneration, atrial natriuretic peptide, neuroprotection, Parkinson’s disease

## Abstract

In the last decades increasing evidence indicated a crucial role of the Wnt/β-catenin signaling in development of midbrain dopaminergic (mDA) neurons. Recently dysregulation of this pathway has been proposed as a novel pathomechanism leading to Parkinson’s disease (PD) and some of the molecules participating to the signaling have been evaluated as potential therapeutic targets for PD. Atrial natriuretic peptide (ANP) is a cardiac-derived hormone having a critical role in cardiovascular homeostasis. ANP and its receptors (NPRs) are widely expressed in mammalian central nervous system (CNS) where they could be implicated in the regulation of neural development, synaptic transmission and information processing, as well as in neuroprotection. Until now, the effects of ANP in the CNS have been mainly ascribed to the binding and activation of NPRs. We have previously demonstrated that ANP affects the Wnt/β-catenin signaling in colorectal cancer cells through a Frizzled receptor-mediated mechanism. The purpose of this study was to investigate if ANP is able to exert neuroprotective effect on two *in vitro* models of PD, and if this effect could be related to activation of the Wnt/β-catenin signaling. As cellular models of DA neurons, we used the proliferating or RA-differentiated human neuroblastoma cell line SH-SY5Y. In both DA neuron-like cultures, ANP is able to positively affect the Wnt/β-catenin signaling, by inducing β-catenin stabilization and nuclear translocation. Importantly, activation of the Wnt pathway by ANP exerts neuroprotective effect when these two cellular systems were subjected to neurotoxic insult (6-OHDA) for mimicking the neurodegeneration of PD. Our data support the relevance of exogenous ANP as an innovative therapeutic molecule for midbrain, and more in general for brain diseases for which aberrant Wnt signaling seems to be involved.

## Introduction

Parkinson’s disease (PD) is a neurodegenerative disorder characterized by a progressive degeneration of mDA neurons in the *substantia nigra* (Hornykiewicz, 1975). The progressive loss of dopamine levels underlays most of the motor symptoms associated with the disease including resting tremors, rigidity, bradykinesia and postural instability along with progressive impairment of autonomic, cognitive, and mood functions (Hirsch et al., 2013). Until now, the exact molecular mechanisms underlying the onset of PD are still unknown, even if genetic and environmental factors, leading to neuroinflammation, oxidative stress, mitochondrial dysfunction, alteration in neurotransmitter receptors (Xu et al., 2012; Kaur et al., 2017), seem to be possible triggers. Currently, there is no cure for PD, and treatments for this disease mostly consist in pharmacotherapy to restore striatal dopamine levels, that only temporary reduce symptoms (Olanow and Schapira, 2013).

In this context, deepening of knowledge on molecular mechanisms underlying PD insurgence and progression is crucial for discovering innovative molecular targets useful for developing more effective therapeutic strategies. In the last years, numerous studies have been published, that aimed to identify innovative PD biomarkers, also useful as therapeutic targets, and to demonstrate the neuroprotective role of endogenous and exogenous molecules (Chen et al., 2015; Price et al., 2015; Wang et al., 2017; Zou et al., 2017)

The Wnt pathway has a key role in a large part of biological processes and regulates, in the CNS, all aspects of neuronal functions including differentiation, synapse formation, neurogenesis and neuroprotection (Inestrosa and Arenas, 2010; Zhang et al., 2011; Harrison-Uy and Pleasure, 2012; Salinas, 2012). In the last decades increasing evidence indicated a crucial role of Wnt/β-catenin signaling in the development of mDA neuron (Prakash et al., 2006; Prakash and Wurst, 2006) and more recently dysregulation of this pathway has been proposed as a novel pathomechanism leading to PD (L’Episcopo et al., 2012, 2013, 2014; Berwick and Harvey, 2014). In intact midbrain, Wnt ligands, and in particular those belonging to Wnt1 class, bind to the Fzd1 and to the LRP5 or LRP6 co-receptors (“Wnt-ON” state) (L’Episcopo et al., 2014), and this event leads to activation and membrane recruitment of the phosphoprotein Dvl. Activated Dvl inhibits the β-catenin destruction complex - through recruitment of Axin at the plasma membrane and induction of the Akt-mediated inactivation of GSK-3β via Ser9 phosphorylation (Fukumoto et al., 2001) – and causes β-catenin stabilization and cytosolic accumulation. Stabilized β-catenin translocates to the nucleus, where it acts as a co-activator for T-cell factor/lymphoid enhancer factor1(TCF/LEF1)-mediated transcription and regulates the expression of Wnt target genes involved in mDA neuron survival/plasticity (L’Episcopo et al., 2014), thus maintaining the integrity of mDA neurons. β-catenin, the most important mediator of the canonical Wnt pathway (Moon et al., 2004), can also behave as a defense molecule against oxidative stress or co-activate nuclear receptors implicated in the maintenance/protection of DA neurons (L’Episcopo et al., 2011a). Neurotoxic agents including PD neurotoxins (6-OHDA, MPTP/MPP+), oxidative stress, aging, or growth factor deprivation can antagonize the Wnt/β-catenin signaling (“Wnt OFF” state) in DA neurons. In this state, up-regulation of active GSK-3β results in the phosphorylation and rapid degradation of β-catenin and increases DA neuron vulnerability, degeneration, and apoptosis (L’Episcopo et al., 2014). Therefore, targeting Wnt signaling could be an effective strategy for neuroprotection/repair in PD (Parish and Arenas, 2007; L’Episcopo et al., 2011a, 2014; Arenas, 2014; Dai et al., 2014; Serafino et al., 2017).

Atrial natriuretic peptide (ANP) is a 28aa peptide that belongs to a family of cardiac and vascular-derived hormones having a critical role in cardiovascular homeostasis mainly by regulating blood volume and pressure (Wilkins et al., 1997; Levin et al., 1998). In the last decade, the new capacity demonstrated for ANP of inhibiting tumor growth both *in vitro* and *in vivo* (Vesely, 2009) has made this peptide an attractive molecule also for anticancer therapy (Vesely, 2012; Serafino and Pierimarchi, 2014). ANP, originally identified in the heart and peripheral tissues, has been also detected in rodent and human brain (Morii et al., 1985; McKenzie et al., 1994). ANP, as well as the other two major components of the natriuretic peptide (NP) family, the brain natriuretic peptide (BNP) and the C-type natriuretic peptide (CNP), together with their receptors (NPRs), are broadly expressed in mammalian CNS and growing evidence indicates that they could be implicated in the regulation of neural development, synaptic transmission and information processing, as well as in neuroprotection (Quirion, 1989; Cao and Yang, 2008; Prado et al., 2010; Mahinrad et al., 2016). Interestingly, it has been reported that ANP inhibits apoptosis induced by serum deprivation in PC12 cells (Fiscus et al., 2001) and that this natriuretic peptide is able to increase TH mRNA and intracellular dopamine levels both *in vitro* and *in vivo* (Takekoshi et al., 2000; Kuribayashi et al., 2006). It has been also shown that pre-treatment with ANP protects NG108-15 cells, a cholinergic-neuron-like cell line, against nitric oxide-induced apoptosis (Cheng Chew et al., 2003), and that this natriuretic peptide has neuroprotective effect against N-methyl-D-aspartate-induced toxicity in rat retinal neurons, probably by the activation of dopamine D1 receptors (Kuribayashi et al., 2006). Until now, the effects of ANP and the other two NPs in the CNS have been mainly ascribed to the binding and activation of NPRs (Cao and Yang, 2008; Mahinrad et al., 2016).

ANP is synthesized as an inactive precursor (pro-ANP) that is converted to the mature active peptide after proteolytic cleavage by the membrane-associated serine protease Corin, which extracellular region encloses the two Frizzled-like cysteine-rich domains Fzd1 and Fzd2, receptors for the Wnt signaling (Knappe et al., 2004; Chan et al., 2005). Corin has been initially found in the heart (Yan et al., 2000), but recent works demonstrated that, in the developing brain, it is specifically expressed on DA progenitor cells located in the floor plate, and is used as marker useful to enrich dopaminergic progenitors in studies on regenerative medicine for PD treatment (Doi et al., 2014; Kikuchi et al., 2017). We have recently demonstrated that ANP influences the Wnt/β-catenin signaling cascade through a Frizzled receptor-mediated mechanism possibly relying on the direct interaction between the Wnt receptor and ANP (Serafino et al., 2012; Serafino and Pierimarchi, 2014). In this study we investigated whether ANP is able to exert neuroprotective effect on two *in vitro* models of PD, and if this effect could be associated with activation of the Wnt/β-catenin pathway. Even if reliable cellular systems to study pathophysiological mechanisms of PD and to test innovative therapeutics are limited, the human neuroblastoma cell line SHSY5Y, proliferating or differentiated by retinoic acid (RA), is currently the most used *in vitro* models of DA neurons (Cheung et al., 2009; Lopes et al., 2010, 2017; Xie et al., 2010). Therefore, for this study we used the SHSY5Y in basal conditions (wild type, SHSY5Ywt) or RA-differentiated (SHSY5Ydiff) as DA neuron-like cells, that were subjected to neurotoxin insult by 6-OHDA for mimicking the neurodegeneration of PD. Both cellular systems have been preliminarily characterized and compared for their phenotypical features and for susceptibility to 6-OHDA, since the current data reported in the literature about which of the two systems is the most suitable model for studying the molecular and cellular mechanisms underlying the pathophysiology of PD are conflicting (Cheung et al., 2009; Lopes et al., 2010, 2017; Xie et al., 2010).

## Materials and Methods

### Cell Cultures and Treatments

SHSY5Y cell line was obtained from the American Type Culture Collection (ATCC, Manassas, VA, United States) and was validated by the purchaser cell bank. Cells were grown as monolayer in Eagle’s Minimum Essential Medium (α-MEM) plus HAM’s F12 (1:1), supplemented with 10% heat-inactivated Fetal Bovine Serum (FBS), L-glutamine (2 mM), penicillin (100 IU/ml) and streptomycin (100 μg/ml) and cell cultures were maintained at 37°C, in a humidified atmosphere of 5% CO_2_. For passaging, cells were detached from culture flasks with 0.05% trypsin and 0.002% EDTA solution. All media and supplements for cell cultures were acquired from Hyclone (Logan, UT, United States).

For evaluating the responsiveness to Wnt signaling-affecting molecules, exponentially growing SHSY5Y (SHSY5Ywt) cells were seeded at densities ranging from 4 × 10^4^/cm^2^ and 6 × 10^4^/cm^2^ and cultured for 24 h prior to treatments. Cells were treated with 100 nM ANP (PeptaNova, Sandhausen, Germany), a concentration selected as the lowest effective and not toxic dose in preliminary dose-response experiments (Supplementary Figure S1), for times ranging from 3 to 24 h. As positive control of Wnt signaling activation, the treatment with Wnt1a 100 ng/ml (ENZO life Science) was used.

The differentiated phenotype (SHSY5Ydiff) was obtained based on protocols previously reported by other Authors (Lopes et al., 2010, 2017). In detail, SHSY5Y cells were seeded at density of 4 × 10^4^/cm^2^ and maintained in culture for 24 h to obtain monolayer at about 75% confluence. Cells were then treated with 10 μM retinoic acid (RA) in absolute ethanol and maintained in low serum medium (1% FBS) for a total of 9 days. To replenish RA, the differentiating agent was added at day 2, day 5, and day 8 after seeding, by replacing cell culture medium with new fresh medium without detaching the cell monolayer, and at day 10 the differentiated cultures were used for the experiments or analyzed. ANP treatment on SHSY5Ydiff was performed as described above for SHSY5Ywt cells.

For mimicking the neurodegeneration of PD, SHSY5Ywt and SHSY5Ydiff cells were exposed for 24 h to 50 and 100 μM of 6-OHDA (prepared in 0.1% ascorbic acid in DMSO), respectively. These concentrations have been selected as the lowest effective doses in preliminary dose-response experiments (Supplementary Figure S2), in which we tested the susceptibility of the two different cellular systems to the neurotoxin, by using concentration ranging from 25 to 400 μM. For assessing the neuroprotective ability of the natriuretic peptide, SHSY5Ywt and SHSY5Ydiff cells were pre-incubated with 100 nM ANP 30 min or 24 h prior to the addition of 6-OHDA to cell cultures, and analyzed after additional 24 h for morphological changes, and cell viability, and for the expression levels of β-catenin, DA neuron specific markers and survival factors.

### Evaluation of Cell Morphology, Viability and Growth, and Cell Cycle

Cell morphology were analyzed by phase-contrast microscopy, using the Motic AE31 Trinocular inverted microscope (Motic Asia, Hong Kong). Cell viability was evaluated by Trypan blue dye exclusion method. Mitotic index (MI) evaluation was carried out as previously described (Serafino et al., 2012). Quantitative evaluation of MI was done in a blinded fashion under the LEICA TCS SP5 Confocal Laser Scanning Microscopy (CLSM, Leica Instruments, Mannheim, Germany), by counting a minimum of 300 cells/sample. Results were reported as percentage of cells in mitosis. Cell viability and proliferation analyses were performed on samples in triplicate and results were reported as mean values ± SD. Cell cycle assessment was carried out by cytofluorimetric analysis of DNA content after propidium iodide (PI) staining. Total DNA content was measured using the FACSCalibur flow cytometer (Becton Dickinson, Franklin Lakes, NJ, United States)

### Immunocytochemical Analysis and Confocal Microscopy (CLSM)

For confocal microscopic analyses, SHSY5Ywt or SHSY5Ydiff cells were grown or RA-differentiated on the ibiTreat μ-Slide 4 well (Ibidi GmbH, Germany, cod. 80426). Immunocytochemical analyses were performed on cells fixed with 2% paraformaldehyde (Sigma–Aldrich). After permeabilization with 0.2% Triton X-100 (Sigma–Aldrich), immunofluorescence staining was done using the primary antibodies against β-catenin, Nestin, Tubulin-β3, NeuN and TH, described in **Table 1**. The specificity of each antibody has been preliminary verified by Western blot, by considering the production of a single band matched to the proper molecular weight. Primary antibodies were revealed with Alexa Fluor 488-conjugated anti-mouse or anti-rabbit IgG (Molecular Probes). Samples were analyzed by using the LEICA TCS SP5 confocal microscope.

**Table 1:** List of antibodies used for immunocytochemical and Western blot analyses.

Antigen	Host	Cat. #	Method/s	Working dilution	Supplier
GSK-3αβ	Rabbit (monoclonal)	ab16667	WB	1:1000	Cell signaling (Boston, MA, United States)
pGSK -3β^Ser9^ (inactive form)	Rabbit (monoclonal)	9323	WB	1:1000	Cell signaling (Boston, MA, United States)
β-catenin	Mouse (monoclonal)	610154 (Clone 14)	IF WB	1:250 1:3000	BD Transduction Labs (Palo Alto, CA, United States)
pβ-catenin^Ser33/37/Thr4^ (preliminary to degradation)	Rabbit (polyclonal)	9561	WB	1:1000	Cell signaling (Boston, MA, United States)
GAPDH	Rabbit (polyclonal)	Sc-25778	WB	1:20000	Santa Cruz Biotechnology, Inc.
AKT	Rabbit (polyclonal)	9272	WB	1:1000	Cell signaling (Boston, MA, United States)
pAKT^Thr308^ (active form)	Rabbit (polyclonal)	9275	WB	1:1000	Cell signaling (Boston, MA, United States)
c-Myc	Rabbit (monoclonal)	Ab32072	WB	1:10000	Abcam (Cambridge, MA, United States)
β-actin	Mouse (monoclonal)	A5441 (Clone AC-15)	WB	1:10 000	Sigma–Aldrich (St. Louis, MO, United States)
Tyrosine hydroxylase	Rabbit (polyclonal)	2792	IF WB	1:100 1:1000	Cell signaling (Boston, MA, United States)
NeuN	Mouse (monoclon)	Ab104224	IF WB	1:500 1:5000	Abcam (Cambridge, MA, United States
DJ-1	Rabbit (polyclonal)	AB9212	WB	1:5000	Millipore
Nestin	Rabbit (monoclonal)	Ab105389	IF WB	1:100 1:1000	Abcam (Cambridge, MA, United States
Frizzled-1	Rabbit (polyclonal)	Sc-130758	WB	1:200	Santa Cruz Biotechnology, Inc.
Nurr1	Mouse (monoclonal)	Sc-81345	WB	1:200	Santa Cruz Biotechnology, Inc.
Tubulin-β3	Mouse (monoclonal)	T8660	WB IF	1:400 1:50	Sigma–Aldrich (St. Louis, MO, United States)

*IF, immunofluorescence; WB, Western blot*.

### Western Blot (WB) Analysis

After washing with ice-cold PBS, cells were lysed using a 50 mM Tris-HCl buffer at pH 8.0. Specifically, the buffer solution contained 150 mM of NaCl, 1% of NP-40, 10% of glycerol, 0.1 mM of EGTA, 0.5 mM of EDTA, 50 mM of NaF, 1 mM of Na3OV4, and a protease inhibitor cocktail (Sigma–Aldrich). Lysates were clarified by centrifugation and proteins were quantified using the Bradford reagent (Bio-Rad, Segrate, Italy). 15–20 μg of each cell extract was separated using SDS/PAGE ranging from 8 to 12%, and then transferred to nitrocellulose membrane (Hybond, Amersham GE Healthcare). Membranes were incubated for 1 h at room temperature with 5% BSA in Tris-buffered saline-Tween (TBS-T; 0.2 M Tris, 1.37 M NaCl, pH 7.6, and 0,05% Tween-20), probed with the specific antibodies reported in **Table 1**. Primary antibodies were revealed with peroxidase-conjugated secondary antibody (BioRad, Richmond, CA, United States). Densitometric analysis was done using the ImageJ processing program^1^. Values, normalized to β-Actin or GAPDH, were reported as uncalibrated Optical Density (OD) or as fold *vs* untreated control. Data were from at least three independent experiments and presented as the mean ± SD.

### Quantitative Reverse Transcription Polymerase Chain Reaction (RT-qPCR)

Transcription levels of β-catenin (β-cat), LEF1 and TH genes were assessed by qPCR in untreated controls and in SHSY5Ywt cells challenged with 100 nM ANP for times ranging from 3 to 24 h. Total RNA was extracted using TRIzolTM Reagent (Invitrogen, Carlsbad, CA, United States), and treated with RNase-free DNase (Promega), according to the manufacturer’s instructions. After extraction with a mix of Phenol:Chloroform:Isoamyl Alcohol (25:24:1; Invitrogen) and precipitation with ethanol, 1 μg of total RNA was reverse-transcribed using random primers with ImProm II Reverse Transcription system (Promega) according to the instructions of the manufacturer. qPCR was performed with iTaq Universal SYBR Green Supermix (Bio-Rad) using 250 nM of the specific primers, by the CFX Connect Real-Time PCR Detection System (Bio-Rad). Sequences of primers used are reported in **Table 2**. The amplification conditions were: 1 min at 95°C, 40 cycles of 10 s at 95°C and 40 s at 60°C. Cq values were determined from the system software using ‘single threshold’ mode. The relative expression level for each gene were calculated from these Cqs using experimentally determined amplification efficiencies, and then normalized for the reference gene GAPDH. Results were reported as fold of induction *vs* untreated control. In all experiments each sample was analyzed in triplicate, and no-template controls and no-reverse transcription controls were inserted.

**Table 2:** Sequences of primers used quantitative reverse transcription polymerase chain reaction (qPCR).

Gene name	Accession number	Forward sequence	Reverse sequence
CTNNB1 (β-catenin, ID: 1499)	NM 001904.3	GTCTGAGGACAAGCCACAAG	CCCTGGGCACCAATATCAAG
LEF1 (ID: 51176)	NM 016269.4	AATGAGAGCGAATGTCGTTGC	GCTGTCTTTCTTTCCGTGCTA
TH (ID: 7054)	NM 000360.3	TGTCCACGCTGTACTGGTTC	TCTCAGGCTCCTCAGACAGG
GAPDH (ID:2597)	NM 002046.5	CCACATCGCTCAGACACCAT	ATGTAAACCATGTAGTTGAGG

### Statistical Analysis

Statistical analysis was performed using the two-tailed Student’s *t*-test, and *p* < 0.05 was considered as statistically significant. All data were from at least three independent experiments and presented as means ± SD.

## Results

### Phenotypical Features of SHSY5Ywt Cells

**FIGURE 1:**
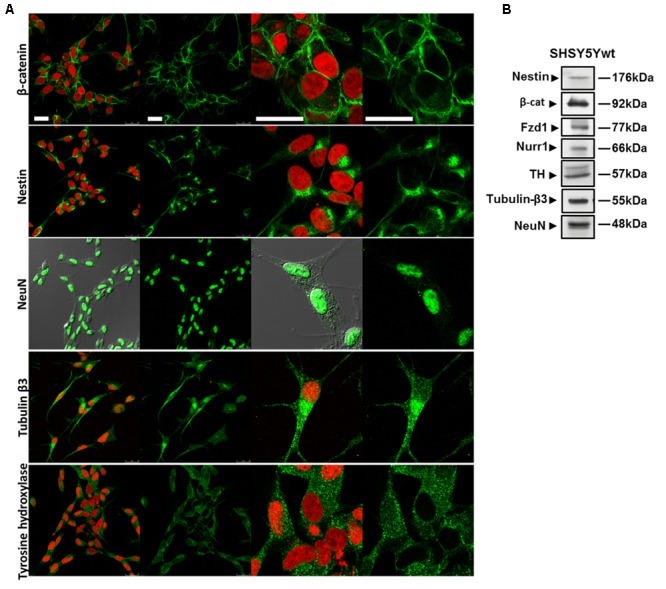
Phenotypical characterization of proliferating SHSY5Y cells (SHSY5Ywt). **(A)** Confocal microscopic images showing the intracellular distribution of the neuronal markers Nestin, Tubulin-β3 and NeuN, of the DA neuron specific marker TH, and of the most important mediator of the canonical Wnt signaling β-catenin. For Tubulin-β3, Nestin, TH, and β-catenin immunostaining, cell nuclei were counterstained with propidium iodide (PI, red hue); for NeuN, cell morphology was visualized by differential interference contrast (DIC). For each marker, both single staining and merged images with PI or DIC are shown. Bars: 25 μm. **(B)** Western blot analysis of expression levels of Nestin, Tubulin-β3, NeuN, TH, Fzd1 receptor and β-catenin in total cell lysates from SHSY5Ywt cells.

SHSY5Y cells in basal condition (SHSY5Ywt) have been characterized for the expression and intracellular distribution not only of neuronal markers (Nestin, Tubulin-β3, NeuN) and of the DA neuron specific markers TH and Nurr1, but also of two crucial molecules participating to the Wnt/β-catenin signaling, the Fzd1 receptor and β-catenin (**Figure 1**). Confocal microscopy (**Figure 1A**) and Western blot analysis (**Figure 1B**) showed that SHSY5Ywt cells expressed Tubulin-β3 and NeuN (specific for mature neurons) as well as TH and Nurr1 (specific for DA neurons), together with detectable levels of Nestin (neural Stem/Progenitor cell marker). This confirms that SHSY5Ywt cells possess the features of dopaminergic neurons and are appropriate for studying neurotoxicity or neuroprotection in experimental PD, as also reported by others (Levites et al., 2003; Cheung et al., 2009; Lopes et al., 2010, 2017; Xie et al., 2010). Importantly, Fzd1 receptor and β-catenin are also clearly expressed (**Figure 1B**), with β-catenin that localized almost exclusively at the cell membrane (**Figure 1A**, upper panels), and this indicated that SHSY5Ywt cells were potentially responsive to molecules affecting the Wnt pathway.

### Setting Up and Characterization of RA-Differentiated SHSY5Y Cells (SHSY5Ydiff)

**FIGURE 2:**
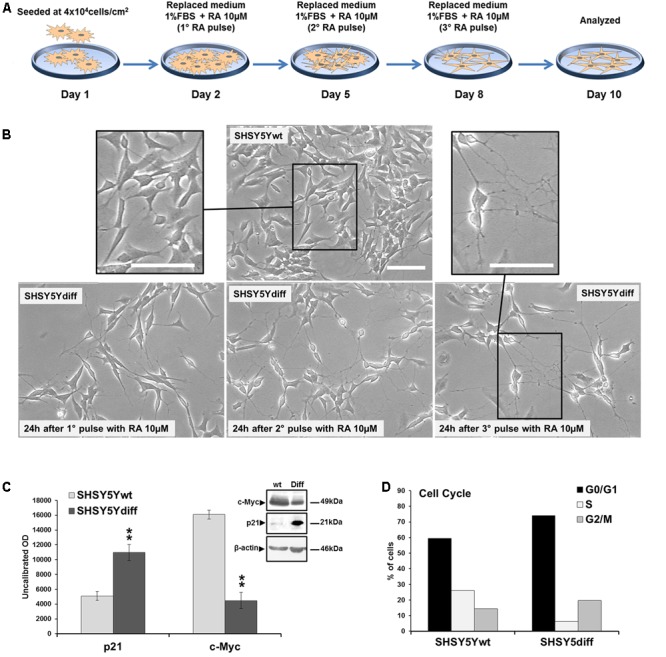
Phenotypical characterization of RA-differentiated SHSY5Y cells (SHSY5Ydiff). **(A)** Resuming scheme of the protocol used for the RA-induced differentiation of SHSY5Y cells. SHSY5Ydiff cells were obtained through a 9-days differentiation process. Cells were seeded at a density of 4 × 10^4^ cells/cm^2^, and after 24 h (day 2), when the confluence of the monolayer was about 75%, were subjected to the 1st pulse with 10 μM RA, by replacing the culture medium with fresh medium containing RA and low serum (1% FBS). The 2nd and the 3rd pulses were performed at day 5 and 8, respectively, by replacing the medium to replenish RA, and at day 10 cells were analyzed or used for the experiments. **(B)** Phase contrast microscopy of SHSY5Ydiff cells 24 h after the 1st, 2nd, and 3rd pulses of 10 μM RA, showing the ongoing of neuronal differentiation with neurite outgrowth. Bar: 100 μm. **(C)** WB and densitometric analyses of the expression levels c-Myc and p21 in SHSY5Ydiff *vs* SHSY5Ywt. **(D)** Cytofluorimetric analysis of DNA content in SHSY5Ywt and SHSY5Ydiff cells. Values from densitometric analyses were normalized to β-actin. Significance SHSY5Ydiff *vs* SHSY5Ywt (Student’s *t*-test): ^∗∗^, *p* < 0.01; the mean ± SD; *n* = 4.

**FIGURE 3:**
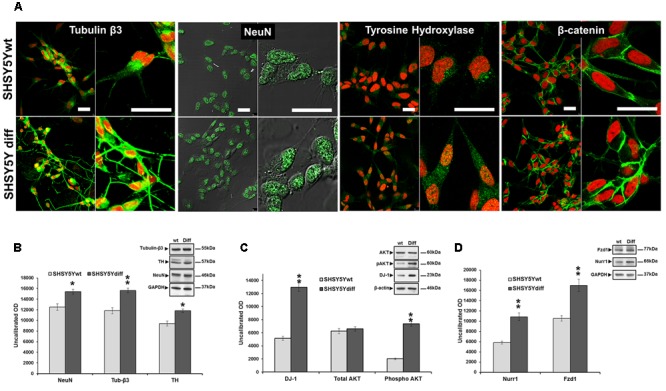
Comparative analyses of phenotypical features in SHSY5Ywt and SHSY5Ydiff cells. **(A)** Confocal microscopy showing the intracellular distribution of Tubulin-β3, NeuN, TH and β-catenin in basal and RA-differentiated SHSY5Y cultures. For Tubulin-β3, TH, and β-catenin immunostaining, cell nuclei were counterstained with propidium iodide (red hue); for NeuN, cell morphology was visualized by differential interference contrast (DIC) and merged images NeuN/DIC are shown. Bars: 25 μm. **(B–D)** WB and densitometric analyses of the expression levels of Tubulin-β3, NeuN and TH, Nurr1 (neuronal and DA neuron specific markers), DJ-1 and phospho-Akt^T308^ (neuronal survival factors involved in neuroprotection against oxidative stress), and Fzd1 (Wnt pathway related molecule), in SHSY5Ywt and SHSY5Ydiff cells. Values from all densitometric analyses were normalized to β-actin or GAPDH. Significance SHSY5Ydiff *vs* SHSY5Ywt (Student’s *t*-test): ^∗^*p* < 0.05; ^∗∗^, *p* < 0.01; the mean ± SD; *n* = 3.

SHSY5Ydiff cells were obtained as described in Section “Materials and Methods” and schematized in **Figure 2A**. Even if proliferating and RA-differentiated SHSY5Y cells have long been used for studies in neuroscience as models of DA neurons, data reported in literature about which of the two systems is the most suitable model for investigating the molecular and cellular mechanisms underlying the pathophysiology of PD are often inconsistent (Cheung et al., 2009; Lopes et al., 2010, 2017; Xie et al., 2010). For this reason, both cellular systems have been preliminarily characterized and compared for their morphological and phenotypical features (**Figures 2, 3**). RA treatment for a total of 9 days gave rise to cells exhibiting neuronal differentiation with neurite outgrowth (**Figure 2B**), low proliferation rate, as demonstrated by lower levels of c-Myc (**Figure 2C**), and block in G1 phase, as demonstrated by cytofluorimetric analysis (**Figure 2D**) and by higher levels of p21 (**Figure 2C**), than those recorded in the untreated SHSY5Y cells. SHSY5Ydiff cells were then compared with SHSY5Ywt for the neuronal markers (Tubulin-β3, NeuN), DA neuron specific markers (TH, Nurr1), neuronal survival factors involved in neuroprotection against oxidative stress (DJ-1 and pAkt^T308^, the phosphorylated active form of AKT), and Wnt pathway related molecules (Fzd1 and β-catenin) (**Figure 3**). Confocal microscopy (**Figure 3A**) and Western blot analysis (**Figures 3B–D**) showed that SHSY5Ydiff cells, expressed higher levels of Tubulin-β3, NeuN, TH and Nurr1 (1.32-fold, 1.23-fold, 1.25-fold, and 1.9-fold of increment, respectively), compared with SHSY5Ywt, indicating that the RA-differentiated cellular system was phenotypically more cognate to mature dopaminergic neurons than the not differentiated one. Moreover, expression levels of the survival factors DJ-1 and active (phosphorylated) AKT were also considerably increased in SHSY5Ydiff as compared with SHSY5Ywt (2.5-fold and 3.7-fold of increment, respectively) (**Figure 3C**). In both cellular systems β-catenin localized almost exclusively at the cell membrane (**Figure 3A**), while Fzd1 receptor was expressed at higher levels (**Figure 3D**) in SHSY5Ydiff compared to SHSY5Ywt (1.6-fold of increment). This indicated that SHSY5Ydiff cells could be potentially responsive to molecules affecting the Wnt pathway, similarly to SHSY5Ywt.

### Assessment of Responsiveness of SHSY5Ywt and SHSY5Ydiff to Wnt Signaling-Affecting Molecules

In order to verify whether the treatment with the natriuretic peptide affected the Wnt signaling also in these cellular systems as previously demonstrated for other cells (Serafino et al., 2012; Skelton et al., 2013a,b; Vesely, 2013), SHSY5Ywt neuroblastoma cells were treated for 24 h with ANP in a preliminary dose-response experiment (Supplementary Figure S1), for selecting the lowest effective and not toxic dose to be used. β-catenin nuclear translocation and the concomitant increase in its expression have been used as main markers of Wnt pathway activation. The concentration of 100 nM ANP has been selected as the lowest dose inducing nuclear β-catenin translocation (Supplementary Figure S1A), cell proliferation arrest with a decrement of mitotic index (MI, Supplementary Figure S1B), increased levels of total β-catenin and a concomitant decrease on β-catenin phosphorylation at T41/S45 (decreased β-catenin degradation; Supplementary Figure S1C). The effects of 100 nM ANP on β-catenin intracellular distribution and degradation were then analyzed in time course experiments and compared with those of the Fzd1 ligand Wnt1a, that specifically triggers the Wnt pathway and has been demonstrated to exert protective effects on SHSY5Y cells (Wei et al., 2013). Hence, 100 ng/ml Wnt1a [concentration utilized by other Authors on SHSY5Y cells (Wei et al., 2013)] was used as positive control of Wnt signaling activation.

**FIGURE 4:**
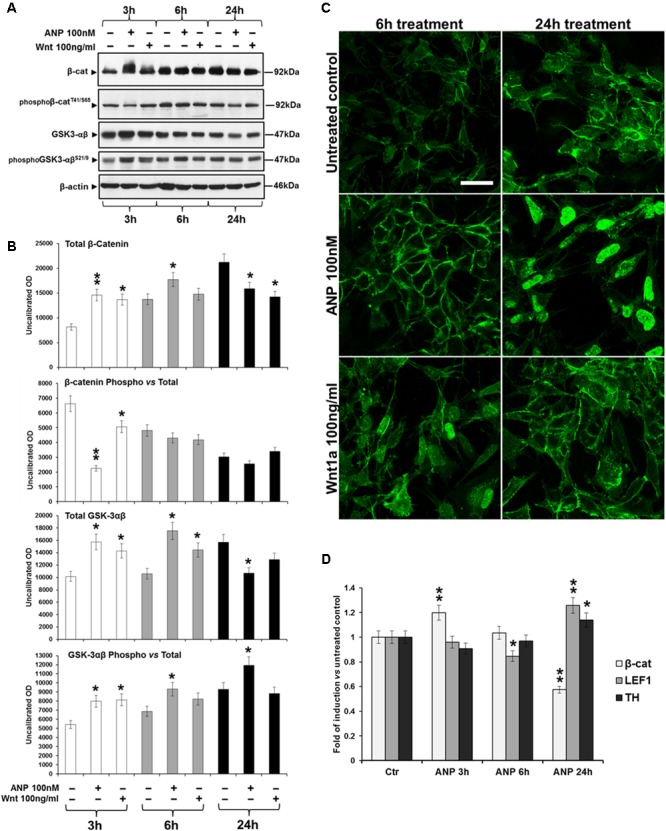
Atrial natriuretic peptide (ANP) modifies β-catenin intracellular distribution and inhibits β-catenin degradation in SHSY5Ywt cells. **(A)** WB analyses of total and phosphorylated β-catenin (pβ-catenin^T41/S65^), and of total and phosphorylated GSK-3β (pGSK-3β^Ser9^) levels in total cell lysates from ANP treated SHSY5Ywt cells, compared to that from untreated control and Wnt1a treated samples; analyses were performed after 3, 6, and 24 h of treatment. **(B)** Results from the densitometric analysis of total and phosphorylated β-catenin and GSK-3β, performed using the ImageJ processing program [http://rsbweb.nih.gov/ij/]; values were normalized to β-actin, for total β-catenin and GSK-3β, or *vs* the total levels of each protein for the phosphorylated forms. Results are the mean from three independent experiments. Significance *vs* untreated relative control: ^∗^*p* < 0.05; ^∗∗^*p* < 0.01; the mean ± SD; *n* = 3. **(C)** Confocal microscopic images showing the intracellular distribution of β-catenin in control and ANP or Wnt1a treated cells after 6 and 24 h of culture. Bar: 25 μm. **(D)** Effect of ANP on the transcription of β-catenin (β-cat), LEF1 and TH genes in SHSY5Ywt cells. Cells were treated with 100 nM ANP and processed for RT-qPCR analysis after 3, 6, and 24 h of treatment. Significance *vs* control (Ctr): ^∗^*p* < 0.05; ^∗∗^*p* < 0.01; the mean ± SD; *n* = 3.

In SHSY5Ywt cells, WB analysis (**Figures 4A,B**) showed that ANP, similarly to Wnt1a, induces a significant increase of total β-catenin (*p* = 0.0098 *vs* untreated control), already after 3 h of treatment, and a concomitant decrease of β-catenin phosphorylation at Thr^41^ (pβ-catenin ^Thr41^), one of the molecular event upstream β-catenin ubiquitination and degradation by the proteasome. Moreover, the decrease of pβ-catenin correlated to an increase of GSK phosphorylation at Ser9 (pGSK-3β^Ser9^), the inactive form of this enzyme (Stambolic and Woodgett, 1994) deputed to β-catenin phosphorylation, as also recorded in Wnt1a treated cells. CLSM observation (**Figure 4C**) showed that ANP also caused a redistribution of β-catenin from cell-cell junction sites to cytoplasmic and nuclear compartments, between 6 and 24 h of treatment. Taken together, these results indicate that, in SHSY5Ywt cells, ANP positively affects the Wnt signaling by inducing β-catenin stabilization and nuclear translocation. This is further supported by the up-regulation of the Wnt-signaling dependent transcriptional factor LEF 1, as measured by RT-qPCR analysis after 24 h of ANP treatment, when a significant increase of transcription of the Nurr1-regulated gene TH was also recorded (**Figure 4D**). Conversely, at 3 and 6 h of ANP treatment, β-catenin mRNA resulted unmodified or down-regulated (**Figure 4D**), indicating that the high levels of the total protein concomitantly recorded by WB (**Figures 4A,B**) were actually due to a reduction of β-catenin degradation induced by the natriuretic peptide, rather than to a decrease transcription of β-catenin gene.

**FIGURE 5:**
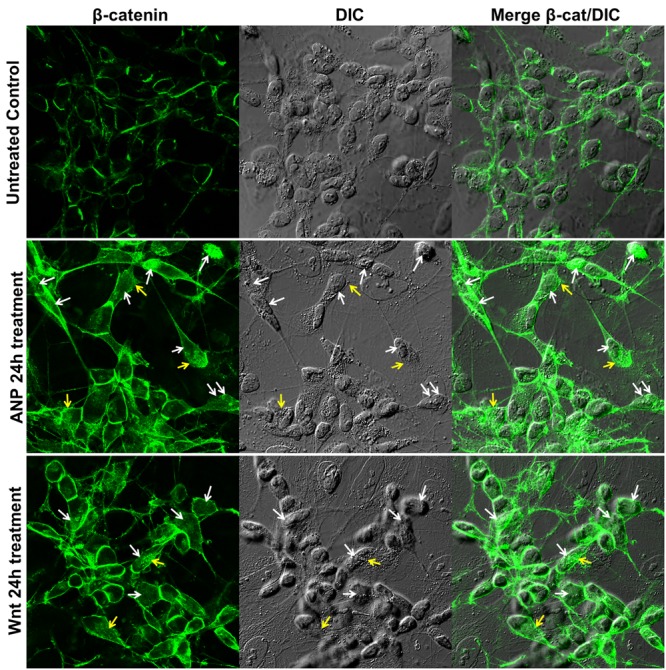
Atrial natriuretic peptide induces β-catenin stabilization and nuclear translocation in SHSY5Ydiff cells. Confocal microscopic images showing the intracellular distribution of β-catenin (green hue) in untreated control and in ANP or Wnt1a treated cells after 24 h of culture. Cell morphology was visualized by differential interference contrast (DIC), and merged images β-catenin/DIC are also shown. White and yellow arrows point to nuclear or cytoplasmic accumulation of β-catenin, respectively. Bar: 25 μm.

SHSY5Ydiff cells were also responsive to treatment with 100 nM ANP, as demonstrate by the β-catenin cytoplasmic accumulation and nuclear translocation observed between 6 and 24 h of treatment, similarly to what observed under treatment with the Fzd1 ligand Wnt1a (**Figure 5**).

### Assessment of 6-OHDA Toxicity on SHSY5Ywt and SHSY5Ydiff Cells

In order to establish the optimal concentration of 6-OHDA to be used in the experiments of neuroprotection, we examined, in a preliminary dose-response test, the susceptibility of both SHSY5Ywt and SHSY5Ydiff to the neurotoxin (Supplementary Figure S2). Proliferative and RA-differentiated SHSY5Y cells were exposed to increasing concentration of 6-OHDA (25, 50, 100, 200, and 400 μM) for 24 h and cytotoxicity was assessed by evaluating cell survival by optical microscopy (Supplementary Figure S2A), cell viability assay (Trypan blue dye exclusion method; (Supplementary Figure S2B) and cell cycle analysis (Supplementary Figure S2C). The concentrations of 50 and 100 μM resulted the lowest effective doses in SHSY5Ywt and SHSY5Ydiff, respectively, that induced detachment of about 50% of cells from the adhering monolayer (Supplementary Figure S2A, red arrows), reduced cell viability by 50–60% (Supplementary Figure S2B) and produced the highest number of apoptotic/necrotic cells by flow cytometry (Supplementary Figure S2A, right panel). The higher dose of neurotoxin selected for SHSY5Ydiff *vs* SHSY5Ywt cells in the dose-response experiments, demonstrated that the RA-differentiated cells obtained by our experimental conditions, were more resistant to 6-OHDA than the not differentiated phenotype. This could be in part due to the higher levels of the survival factors DJ-1 and active AKT recorded by WB in SHSY5Ydiff compared to SHSY5Ywt (**Figure 3C**).

### Neuroprotective Effect of ANP against 6-OHDA Induced Cytotoxicity

**FIGURE 6:**
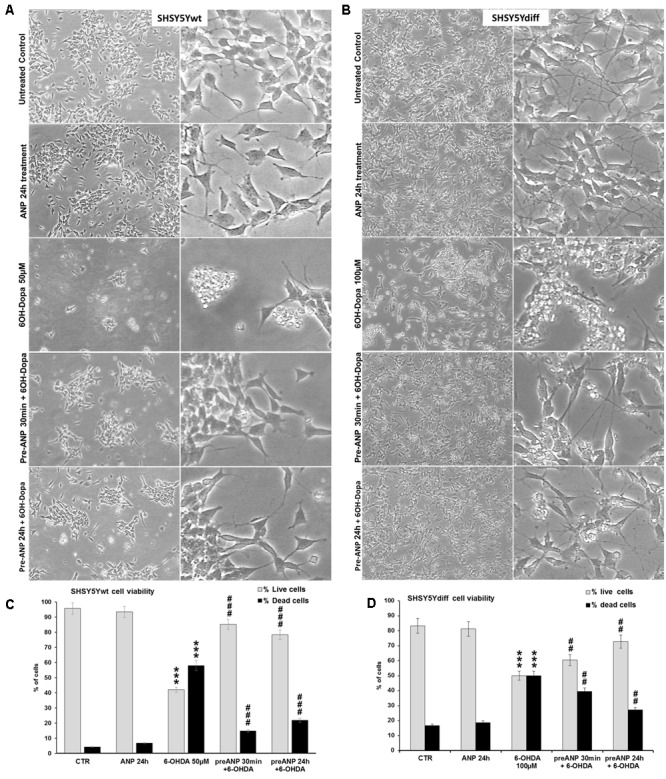
Atrial natriuretic peptide pre-treatment prevents 6-OHDA toxicity on SHSY5Ywt and SHSY5Ydiff cells. **(A,B)** Phase contrast microscopy of SHSY5Ywt **(A)** and SHSY5Ydiff **(B)** cells exposed to 50 and 100 μM of 6-OHDA, respectively, for mimicking the neurodegeneration of PD. To verify the ability of ANP in preventing the 6-OHDA induced cytotoxicity, cells were pre-treated with 100 nM of the natriuretic peptide, 30 min or 24 h prior to 6-OHDA addition to cell culture medium. Original magnification: *left panels*: 10x; *right panels*: 20x. **(C,D)** Cell viability assay performed on SHSY5Ywt **(C)** and SHSY5Ydiff **(D)** cells after 24 h of 6-OHDA treatment by Trypan blue dye exclusion method; results, reported as percentage of live/dead cells, are mean of three independent experiments. **^∗^**Significance *vs* untreated control (Ctr); **^#^**Significance *vs* 6OH-Dopa: ^∗^*p* < 0.05; ^∗∗^*p* < 0.01; ^∗∗∗^*p* < 0.001; the mean ± SD; *n* = 3.

Based on results from the preliminary dose-response test, SHSY5Ywt and SHSY5Ydiff were exposed to 50 and 100 μM of 6-OHDA, for times up to 24 h. To verify the ability of ANP in preventing the cytotoxicity induced by 6-OHDA, cells were pre-treated with 100 nM of the natriuretic peptide, 30 min or 24 h before 6-OHDA addition to cell culture medium. Phase contrast microscopy (**Figures 6A,B**) showed that 6-OHDA treatment drastically decreased the number of adhering cells and destroyed the neurite network in both proliferating and differentiated SHSY5Y cells, while exposure to ANP did not influenced cell adhesion and viability nor neurite integrity. Pre-treatments with 100 nM ANP were able to substantially prevent detachment of cells from the substrate, also partially preserving the neuritic network (**Figures 6A,B**), and significantly (*p* < 0.01) reduced the percentage of dead cells induced by 6-OHDA in both cellular models (**Figures 6C,D**).

**FIGURE 7:**
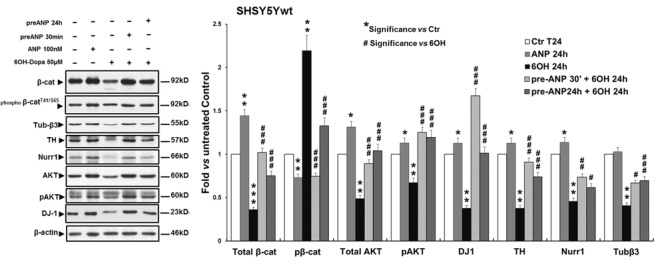
Atrial natriuretic peptide pre-treatment reverses the neurotoxin-induced changes in β-catenin phosphorylation and degradation and in the expression of DA neuron specific markers and survival factors in SHSY5Ywt cells. WB and densitometric analysis of the expression levels of Tubulin-β3, NeuN and TH, Nurr1 (neuronal and DA neuron specific markers), DJ-1 and phospho-Akt^T308^ (neuronal survival factors involved in neuroprotection against oxidative stress), and total and phosphorylated β-catenin (pβ-catenin^T41/S65^,Wnt pathway related molecules), in SHSY5Ywt cells subjected to 24 h 6-OHDA challenging in absence and in presence of 30 min or 24 h pre-treatment with ANP. Values from densitometric analysis were normalized to β-actin, or *vs* the total levels of β-catenin or AKT for the phosphorylated forms. **^∗^**Significance *vs* untreated control (Ctr); **^#^**Significance *vs* 6OH-Dopa: ^∗^*p* < 0.05; ^∗∗^*p* < 0.01; ^∗∗∗^*p* < 0.001; the mean ± SD; *n* = 3.

In SHSY5Ywt cells, WB analysis (**Figure 7**) showed that the challenging with 6-OHDA for 24 h dramatically decreased total β-catenin expression (*p* < 0.001), compared to both untreated control and ANP-treated cells (2.8-fold and 4-fold of decrement, respectively), and concomitantly increased β-catenin phosphorylation and degradation (2.2-fold and 3-fold of increment *vs* untreated control and ANP treated cells, respectively). Moreover, 6-OHDA significantly (*p* < 0.01) reduced the expression levels of the neuronal marker Tubulin-β3 and of the DA neuron specific markers TH and Nurr1 (2.4-fold, 2.6-fold, and 1.8-fold of decrement, respectively) as well as of the survival factors involved in neuroprotection against oxidative stress AKT/phospo-AKT and DJ-1 (2-fold/1.5-fold, and 1.7-fold of decrement, respectively). Pre-treatment with ANP, by inducing β-catenin stabilization, partially or completely restored the expression of all markers analyzed to levels near to those recorded in the untreated control at T0 (**Figure 7** and **Table 3**). It is noteworthy that 24 h of treatment with ANP alone significantly up-regulated the expression levels of the survival and anti-apoptotic factor DJ-1 (**Figure 7**), the product of the PARK3 gene, that has a neuroprotective role against oxidative stress and is dysfunctional or low expressed in PD patients (Bonifati et al., 2003; Canet-Aviles et al., 2004; Martinat et al., 2004; Taira et al., 2004).

**Table 3:** Resuming scheme of neuroprotective effect of ANP pre-treatment on SHSY5Ywt and SHSY5Ydiff cells subjected to 6OH-Dopa insult.

Parameter evaluated	Related function	ANP 100 nM	6OH-Dopa 24 h	Pre-ANP 30 min + 6OH-Dopa	Pre-ANP 24h + 6OH-Dopa
Cell adhesion	Cell integrity	Unmodified	Reduced of ∼50%	Preserved	Preserved
Neuritic network	Cell integrity and functionality	Unmodified	Destroyed	Partially preserved	Partially preserved
Cell viability	Cell survival	Unmodified	Reduced of 50–60%	Significantly recovered (*p* < 0.01)	Significantly recovered (*p* < 0.01)
β-catenin (protein)	Canonical Wnt signaling mediator	Up-regulated (β-cat stabilization)	Down-regulated	Significantly restored near control values (*p* < 0.001)	Significantly restored near control values (*p* < 0.001)
pβ-catenin	Preliminary to β-cat degradation	Down-regulated (decreased β-cat degradation)	Up-regulated (increased β-cat degradation)	Significantly restored near control values (*p* < 0.001)	Significantly restored near control values (*p* < 0.001)
pAKT^Thr308^ (active form)	Survival factor involved in neuroprotection	Up-regulated	Down-regulated in wt Up-regulated in diff	Up-regulated	Up-regulated
DJ1	Survival factor involved in neuroprotection, dysfunctional or low expressed in PD	Up-regulated in wt Unmodified in diff	Down-regulated	Restored near control values	Restored near control values
Nurr1	DA neuron specific marker; expression regulated by nuclear β-cat	Up-regulated	Down-regulated	Significantly restored near control values (p < 0.01)	Significantly restored near control values (p < 0.05)
Tyrosine hydroxylase	DA neuron specific marker; expression regulated by Nurr-1	Up-regulated	Down-regulated	Restored near control values	Restored near control values

**FIGURE 8:**
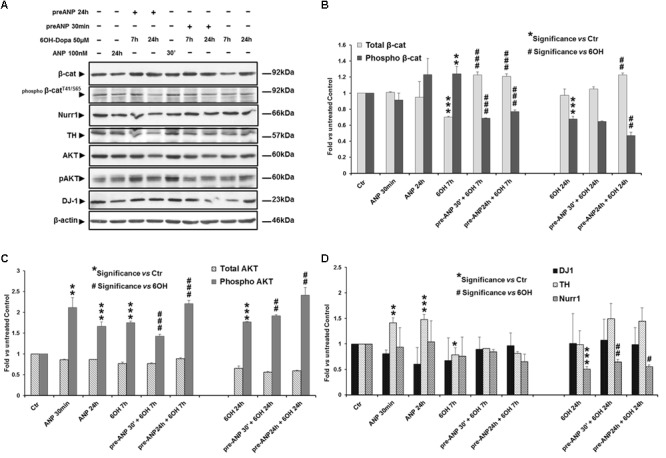
Atrial natriuretic peptide pre-treatment reverses the neurotoxin-induced changes in β-catenin phosphorylation and degradation and in the expression of DA neuron specific markers and survival factors in SHSY5Ydiff cells. WB **(A)** and densitometric **(B–D)** analyses of the expression levels of TH, Nurr1, DJ-1 and phospho-Akt^T308^, and total and phosphorylated β-catenin (pβ-catenin^T41/S65^), in SHSY5Ydiff cells subjected to 7h and 24h 6-OHDA challenging in absence and in presence of 30 min or 24 h pre-treatment with ANP. In the panel of WB **(A)**, the duration of each treatment is reported above the corresponding lane. Values from densitometric analysis were normalized to β-actin, or *vs* the total levels of β-catenin or AKT for the phosphorylated forms. **^∗^**Significance *vs* untreated control (Ctr); **^#^**Significance *vs* 6OH-Dopa: ^∗^*p* < 0.05; ^∗∗^*p* < 0.01; ^∗∗∗^*p* < 0.001; the mean ± SD; *n* = 3.

Similar results were obtained when we used as model of DA neurons the SHSY5Ydiff cells, where the effects induced on the molecular markers were assessed at 7 and 24 h of 6-OHDA treatment (**Figures 8A–D**). In particular, already after 7 h of treatment, the neurotoxin was able to significantly (*p* < 0.001) decrease total β-catenin expression, and concomitantly increase β-catenin phosphorylation and degradation (0.7-fold and 1.24-fold of change *vs* untreated control, respectively) (**Figure 8B**), also reducing, between 7 h and 24 h, the DA neuron specific markers TH and Nurr-1 (**Figure 8D**), while the survival factor and PD-related protein DJ-1 was not significantly modified. Differently to what recorded the proliferating model, in RA-differentiated cells the neurotoxin induces a significant increase in pAKT levels at both times examined (increment: 1.75-fold and 1.77-fold *vs* untreated control, at 7 and 24 h, respectively) (**Figure 8C**), and this could additionally explain the higher resistance to the neurotoxin-induced stress exhibited by the SHSY5Ydiff compared to SHSY5Ywt. Nevertheless, also in SHSY5Ydiff pre-treatment with ANP partially or completely reversed the neurotoxin-induced changes of the markers analyzed already after 7 h of treatment (**Figure 8** and **Table 3**). In particular, at this time point, in ANP pre-treated samples we recorded a significant (*p* < 0.001) increase in total β-catenin and a concomitant decrease in β-catenin phosphorylation and degradation, compared to cells treated with 6-OHDA alone (**Figure 8B**). A significant (*p* < 0.01) increase of Nurr-1 expression was also recorded at 24 h in ANP pre-treated samples. At 24 h, TH, which expression is regulated by Nurr-1 (Sakurada et al., 1999), was also up-regulated in ANP pre-treated samples *vs* 6-OHDA alone, even if the mean values from three independent experiments were not significant (*p* > 0.05; **Figure 8D**). Moreover, in samples pre-treated with ANP for 24 h and subjected to the neurotoxin challenging, we recorded an additional increment in AKT phosphorylation levels compared with 6-OHDA alone, and this further sustains the neuroprotective role exerted by the natriuretic peptide (**Figure 8C**). The neuroprotective efficacy of the natriuretic peptide was also recorded in both SHSY5Ywt and SHSY5Ydiff models subjected to pre-treatment with ANP for 30 min, and this suggests that the molecular events that reinforce cell survival are triggered soon after its addition to culture medium (**Figures 6–8** and **Table 3**).

## Discussion

The knowledge on the role of Wnt/β-catenin signaling in neurodegenerative diseases, and in particular in PD, is still in its infancy, and there are not any ongoing clinical studies specific on this issue, but only data concerning some preclinical therapeutic approaches, performed on cellular and animal models (Serafino et al., 2017). The majority of papers published on this issue aim to demonstrate as the pharmacological activation of β-catenin signaling, by Wnt1 or Wnt1-like agonists (Wei et al., 2013) or by GSK inhibitors (L’Episcopo et al., 2011a; Zhou et al., 2016), has neuroprotective capacity against DA neuron-specific toxins or is able to prevent loss of mDA neurons in the *substantia nigra* and to ameliorate motor symptoms in animal models of PD (L’Episcopo et al., 2011a). It has been very recently reported that the neuroprotective effect of the GSK inhibitors LiCl and SB216763, leading to β-catenin stabilization, is mediated by induction of the orphan nuclear receptor Nurr1 (Zhang et al., 2016), a member of the steroid/thyroid nuclear receptor superfamily that exert different and even antagonistic functions in neuronal cells (Gao et al., 2016). Specifically, Nurr1 is known to have critical roles in the survival and functional maintenance of mDA neurons (Jankovic et al., 2005), and is up-regulation by various neuroprotective agents (Wei et al., 2016). In the last years, numerous evidence suggests that statins, and in particular Simvastatin, are neuroprotective on *in vitro* models of PD and therapeutically helpful for neurological disorders, also including PD (Wolozin et al., 2007; Xu et al., 2013). Even if the mechanisms underlying Simvastatin-induced neuroprotection are not fully understood, it has been recently demonstrated that the effect could be also mediated by up-regulation of Wnt/β-catenin signaling (Robin et al., 2014). Up today, the preclinical data on the neuroprotective capacity of Wnt-related molecules are still limited. Nevertheless, many of the drugs that function as Wnt agonist by targeting the up-stream events of the signaling, such as Fzd receptors ligand, DKK inhibitors and GSK inhibitors, that are currently under preclinical and clinical investigation for cancer therapy, could be hypothetically candidate for developing new therapeutic approaches for PD cure as well as for other neurodegenerative diseases (Serafino et al., 2017).

In the present work we obtained evidence, even with the implicit limitations of an *in vitro* study, that ANP could be a promising Wnt-targeting molecule for PD prevention/therapy. The two cellular models of DA neurons used for this study, the proliferating SHSY5Ywt and the RA-differentiated SHSY5Ydiff, expressed significant levels not only of neuronal and DA neuron specific markers, confirming that they possess the features of mature DA neurons, as previously reported by other authors (Cheung et al., 2009; Lopes et al., 2010, 2017; Xie et al., 2010), but also of crucial molecules participating to the Wnt/β-catenin signaling, and in particular the Fzd1 receptor, indicating that they are hypothetically responsive to molecules affecting upstream events of the canonical Wnt pathway. Indeed, we show that, in both DA neuron-like models, ANP is able to positively affect the Wnt/β-catenin signaling, by inducing β-catenin stabilization and nuclear translocation, similarly and even more efficiently than the Wnt1a - used as positive control of Wnt signaling activation - and that, through this mechanism, it exerts neuroprotective effect when these two cellular systems were subjected to 6-OHDA insult for mimicking the neurodegeneration of PD. As resumed in **Table 3**, in both SHSY5Ywt and SHSY5Ydiff cells pre-treatment with ANP was able to substantially attenuate the effects induced by 6-OHDA on cell integrity and survival, on DA neuron related markers and on survival factors involved in neuroprotection against oxidative stress, even if the PD-related protein DJ-1 was significantly affected only in SHSY5Ywt cells. These effects were significantly associated with β-catenin stabilization (**Table 3**), and this strongly suggests that the ANP-induced neuroprotection against 6-OHDA challenging was strictly related to canonical Wnt signaling activation. The involvement of Wnt/β-catenin pathway activation in the neuroprotective ability of ANP is also sustained by the significant increment in Nurr1 expression in samples pre-treated with the natriuretic peptide before 6-OHDA challenging, recorded after 24 h of treatment in both cellular models (**Table 3**). In fact, it has been very recently reported that β-catenin, after nuclear translocation, binds on the upstream promoter region of Nurr1 and increases its transcription, thus directly regulating Nurr1 gene expression (Zhang et al., 2016).

**FIGURE 9:**
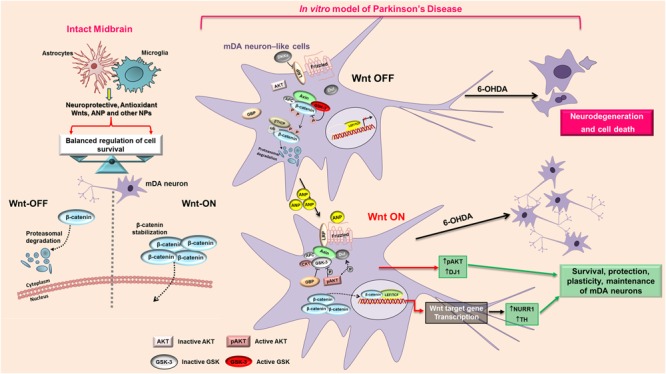
Proposed model of the mechanism that mediates the neuroprotective action of ANP on PD cellular model. In intact midbrain, the balancing between Wnt-ON and Wnt-OFF in mDA is regulated by the microglial/astrocytic component, that produce and release neuroprotective molecules including Wnts, ANP and other NPs. In the *in vitro* model of PD, 6-OHDA antagonizes the Wnt/β-catenin signaling and shifts the balancing toward the “Wnt OFF” state, leading to the up-regulation of active GSK-3β with a consequent phosphorylation and rapid β-catenin degradation, thus increasing cell death of mDA neuron-like cells. ANP, possibly by a direct interaction with the Frizzled receptor, activates the Wnt/β-catenin signaling cascade, induces β-catenin stabilization and nuclear translocation, up-regulates the survival factors pAKT and DJ-1 and activates transcription of the DA neuron markers Nurr1 and TH, thus leading to survival and protection of mDA neurons. APC, adenomatous polyposis coli; AKT, Serine/Threonine Protein Kinase; β-TrCP, E3 ubiquitin ligase; CK1, casein kinase 1; DKK, Dickkopf; Dvl, Disheveled; GBP, GSK3-binding protein; GSK, glycogen synthase kinase; LRP, LDL receptor-related protein; P, phosphorylation; TCF/LEF1, T-cell factor/lymphoid enhancer factor1.

It is noteworthy that, besides the neuroprotective ability demonstrated by the natriuretic peptide, in SHSY5Y proliferating neuroblastoma cells the effects induced by ANP on Wnt signaling caused the inhibition of proliferation, as demonstrated by the MI decrement recorded in cells treated with concentration ≥ 100 nM (Supplementary Figure S1), as well as by the lower levels of the cell growth regulator c-Myc *vs* untreated control (data not shown) similarly to what we have previously demonstrated for colorectal cancer cells (Serafino et al., 2012; Serafino and Pierimarchi, 2014). Interestingly, while in colorectal cancer cells, where the Wnt/β-catenin pathway is constitutively activated, ANP acts by inhibiting the signaling (Serafino et al., 2012), in neuroblastoma cells, in which the pathway is basically not activated, as demonstrated by the localization of β-catenin almost exclusively at the cell membrane, the natriuretic peptide inhibits cell proliferation by functioning as a Wnt pathway activator. This is strongly supported by the up-regulation of the Wnt-signaling dependent transcriptional factor LEF 1, evidenced by RT-qPCR analysis after 24 h of ANP treatment (**Figure 4D**). Thus, ANP seems to be able of acting as a Wnt agonist or antagonist, depending on the cell type and/or pathological situations, and this strongly increases the attractiveness of this natriuretic hormone as an innovative therapeutic approach in a wide range of diseases. Actually, ANP could function as a Wnt modulator capable of successfully reverting aberrant Wnt signaling in different pathological situations, by restoring the balancing between Wnt-OFF and Wnt-ON state. This could also be one of the function in the brain of endogenous ANP, that, together with the two other natriuretic peptides BNP and CNP, is widely expressed in mammalian CNS (Morii et al., 1985; McKenzie et al., 1994), where they seem to have a role in regulating several functions including neural development and neuroprotection (Quirion, 1989; Cao and Yang, 2008; Prado et al., 2010). It is known that the balancing between Wnt-ON and Wnt-OFF in mDA is regulated by the microglial/astrocytic component of the midbrain (L’Episcopo et al., 2011a,b,c, 2014), and astroglial and microglial cells have been reported to produce and release ANP and the other NPs and to express functional NPRs (Prado et al., 2010). In other words, microglial/astrocytic systems seem to be both potential sources and targets of NPs that in turn exert autocrine or paracrine actions on glial cells and on surrounding neurons. It has also been suggested that the NP–NPR system of glial cells participates in neuron-glia communication and could play important roles in the regulation of neuroinflammation and neuroprotection (Prado et al., 2010). Our results strongly suggest that this regulatory role of ANP in the midbrain is possibly mediated not only by an interaction with the NPRs and activation of the cGMP-dependent signaling, as previously reported (Prado et al., 2010; Mahinrad et al., 2016), but also by modulation of Wnt signaling (**Figure 9**), and are encouraging for future studies aimed to deep this issue.

As it concerns the possible mechanism through which ANP triggers the Wnt/β-catenin signaling, we hypothesize that it could involve a Frizzled-related modality, as previously demonstrated for colorectal cancer cells (Serafino et al., 2012), rather that the specific NPR (**Figure 9**). The rationale for this assumption resides in the fact that, the two Frizzled-like cysteine-rich domains, Fzd1 and Fzd2, enclosed in the extracellular region of Corin, are structural domains crucial for pro-ANP processing (Knappe et al., 2004), and this makes reasonable a direct interaction between ANP and the Frizzled receptor. Moreover, it has been reported that SHSY5Y cells did not express a functional NPR-A receptor since 100 nM ANP failed to elicit significant cyclic GMP responses(Forgeur et al., 1999).

In this work we clearly demonstrate, for the first time, that ANP possesses neuroprotective ability on DA neurons by directly modulating the Wnt/β-catenin pathway. We purchase strong *in vitro* evidence that support the relevance of exogenous ANP as an innovative protective/therapeutic molecule for midbrain, and more in general for brain diseases for which aberrant Wnt signaling seems to be the leading pathomechanism. This is particularly stimulating also taking into account that human recombinant ANP (Carperitide) has been already approved by the Ministry of Health and Welfare of Japan (Saito, 2010) as a drug for treatment of heart diseases/dysfunctions and hypertension, and, *de facto*, it has already passed the toxicological screening for the extension of the therapeutic application of this molecule.

Even if further studies, aimed to demonstrate the neuroprotective effectiveness of this natriuretic peptide in animal models of PD, will be required, these results indicate that modulation by ANP of Wnt/β-catenin signaling in neuron cells, and in particular in mDA neurons, could be an innovative approach for PD treatment, and represent a possible starting point for future clinical applications.

## Author Contributions

ACB and EP carried out the setting and characterization of the cellular models, performed the treatments of cell cultures, Western blot analysis and immunocytochemistry, and helped to draft the manuscript. FA performed the experiments of immunofluorescence, cell viability and growth and cell cycle. SR carried out RT-qPCR. MC supervised the RT-qPCR experiments and revised critically the manuscript. GN and GS helped to draft the manuscript and revised it critically. PP performed the cytofluorimentric analysis and revised critically the manuscript. AS designed the study, supervised all experiments, analyzed the data, performed morphological and confocal microscopic analyses, carried out the statistical analysis and draft the manuscript. All authors read and approved the final manuscript.

## Conflict of Interest Statement

The authors declare that the research was conducted in the absence of any commercial or financial relationships that could be construed as a potential conflict of interest.

## Footnotes

1 http://rsbweb.nih.gov/ij/

## Abbreviations

6-OHDA: 6-hydroxy-dopamine
ANP: atrial natriuretic peptide
CLSM: confocal laser scanning microscopy
CNS: central nervous system
DSH: disheveled
Fzd1: Frizzled-1 receptor
GSK-3β: glycogen synthetase kinase-3β
mDA: midbrain dopaminergic
NP: natriuretic peptide
NPRs: natriuretic peptide receptors
Nurr1: nuclear receptor related 1 protein
PD: Parkinson’s disease
TH: tyrosine hydroxylase

## References

[B1] Arenas E. (2014). Wnt signaling in midbrain dopaminergic neuron development and regenerative medicine for Parkinson’s disease. *J. Mol. Cell Biol.* 6 42–53. 10.1093/jmcb/mju001 24431302

[B2] Berwick D. C. Harvey K. (2014). The regulation and deregulation of Wnt signaling by PARK genes in health and disease. *J. Mol. Cell Biol.* 6 3–12. 10.1093/jmcb/mjt037 24115276 PMC4344548

[B3] Bonifati V. Rizzu P. van Baren M. J. Schaap O. Breedveld G. J. Krieger E. (2003). Mutations in the DJ-1 gene associated with autosomal recessive early-onset parkinsonism. *Science* 299 256–259. 10.1126/science.1077209 12446870

[B4] Canet-Aviles R. M. Wilson M. A. Miller D. W. Ahmad R. McLendon C. Bandyopadhyay S. (2004). The Parkinson’s disease protein DJ-1 is neuroprotective due to cysteine-sulfinic acid-driven mitochondrial localization. *Proc. Natl. Acad. Sci. U.S.A.* 101 9103–9108. 10.1073/pnas.0402959101 15181200 PMC428480

[B5] Cao L. H. Yang X. L. (2008). Natriuretic peptides and their receptors in the central nervous system. *Prog. Neurobiol.* 84 234–248. 10.1016/j.pneurobio.2007.12.003 18215455

[B6] Chan J. C. Knudson O. Wu F. Morser J. Dole W. P. Wu Q. (2005). Hypertension in mice lacking the proatrial natriuretic peptide convertase corin. *Proc. Natl. Acad. Sci. U.S.A.* 102 785–790. 10.1073/pnas.0407234102 15637153 PMC545541

[B7] Chen D. Wei X. Zou J. Wang R. Liu X. Xu X. (2015). Contra-directional expression of serum homocysteine and uric acid as important biomarkers of multiple system atrophy severity: a cross-sectional study. *Front. Cell Neurosci.* 9:247. 10.3389/fncel.2015.00247 26217177 PMC4492156

[B8] Cheng Chew S. B. Leung P. Y. Fiscus R. R. (2003). Preincubation with atrial natriuretic peptide protects NG108-15 cells against the toxic/proapoptotic effects of the nitric oxide donor S-nitroso- N-acetylpenicillamine. *Histochem. Cell Biol.* 120 163–171. 10.1007/s00418-003-0568-6 14504961

[B9] Cheung Y. T. Lau W. K. Yu M. S. Lai C. S. Yeung S. C. So K. F. (2009). Effects of all-trans-retinoic acid on human SH-SY5Y neuroblastoma as in vitro model in neurotoxicity research. *Neurotoxicology* 30 127–135. 10.1016/j.neuro.2008.11.001 19056420

[B10] Dai T. L. Zhang C. Peng F. Niu X. Y. Hu L. Zhang Q. (2014). Depletion of canonical Wnt signaling components has a neuroprotective effect on midbrain dopaminergic neurons in an MPTP-induced mouse model of Parkinson’s disease. *Exp. Ther. Med.* 8 384–390. 10.3892/etm.2014.1745 25009587 PMC4079420

[B11] Doi D. Samata B. Katsukawa M. Kikuchi T. Morizane A. Ono Y. (2014). Isolation of human induced pluripotent stem cell-derived dopaminergic progenitors by cell sorting for successful transplantation. *Stem Cell Reports* 2 337–350. 10.1016/j.stemcr.2014.01.013 24672756 PMC3964289

[B12] Fiscus R. R. Tu A. W. Chew S. B. (2001). Natriuretic peptides inhibit apoptosis and prolong the survival of serum-deprived PC12 cells. *Neuroreport* 12 185–189. 10.1097/00001756-200102120-00003 11209918

[B13] Forgeur A. Willems F. Winand J. Robberecht P. Delporte C. (1999). Natriuretic peptide receptors of type A in human neuroblastomas. *Neuroendocrinology* 70 288–294. 10.1159/000054488 10529624

[B14] Fukumoto S. Hsieh C. M. Maemura K. Layne M. D. Yet S. F. Lee K. H. (2001). Akt participation in the Wnt signaling pathway through Dishevelled. *J. Biol. Chem.* 276 17479–17483. 10.1074/jbc.C000880200 11278246

[B15] Gao H. Chen Z. Fu Y. Yang X. Weng R. Wang R. (2016). Nur77 exacerbates PC12 cellular injury in vitro by aggravating mitochondrial impairment and endoplasmic reticulum stress. *Sci. Rep.* 6:34403. 10.1038/srep34403 27679973 PMC5041156

[B16] Harrison-Uy S. J. Pleasure S. J. (2012). Wnt signaling and forebrain development. *Cold Spring Harb. Perspect. Biol.* 4:a008094. 10.1101/cshperspect.a008094 22621768 PMC3385962

[B17] Hirsch E. C. Jenner P. Przedborski S. (2013). Pathogenesis of Parkinson’s disease. *Mov. Disord.* 28 24–30. 10.1002/mds.25032 22927094

[B18] Hornykiewicz O. (1975). Parkinson’s disease and its chemotherapy. *Biochem. Pharmacol.* 24 1061–1065. 10.1016/0006-2952(75)90190-2 239718

[B19] Inestrosa N. C. Arenas E. (2010). Emerging roles of Wnts in the adult nervous system. *Nat. Rev. Neurosci.* 11 77–86. 10.1038/nrn2755 20010950

[B20] Jankovic J. Chen S. Le W. D. (2005). The role of Nurr1 in the development of dopaminergic neurons and Parkinson’s disease. *Prog. Neurobiol.* 77 128–138. 10.1016/j.pneurobio.2005.09.001 16243425

[B21] Kaur K. Gill J. S. Bansal P. K. Deshmukh R. (2017). Neuroinflammation - A major cause for striatal dopaminergic degeneration in Parkinson’s disease. *J. Neurol. Sci.* 381 308–314. 10.1016/j.jns.2017.08.3251 28991704

[B22] Kikuchi T. Morizane A. Doi D. Magotani H. Onoe H. Hayashi T. (2017). Human iPS cell-derived dopaminergic neurons function in a primate Parkinson’s disease model. *Nature* 548 592–596. 10.1038/nature23664 28858313

[B23] Knappe S. Wu F. Madlansacay M. R. Wu Q. (2004). Identification of domain structures in the propeptide of corin essential for the processing of proatrial natriuretic peptide. *J. Biol. Chem.* 279 34464–34471. 10.1074/jbc.M405041200 15192093

[B24] Kuribayashi K. Kitaoka Y. Kumai T. Munemasa Y. Kitaoka Y. Isenoumi K. (2006). Neuroprotective effect of atrial natriuretic peptide against NMDA-induced neurotoxicity in the rat retina. *Brain Res.* 1071 34–41. 10.1016/j.brainres.2005.11.068 16443199

[B25] L’Episcopo F. Serapide M. F. Tirolo C. Testa N. Caniglia S. Morale M. C. (2011a). A Wnt1 regulated Frizzled-1/beta-Catenin signaling pathway as a candidate regulatory circuit controlling mesencephalic dopaminergic neuron-astrocyte crosstalk: Therapeutical relevance for neuron survival and neuroprotection. *Mol. Neurodegener.* 6:49. 10.1186/1750-1326-6-49 21752258 PMC3162575

[B26] L’Episcopo F. Tirolo C. Testa N. Caniglia S. Morale M. C. Cossetti C. (2011b). Reactive astrocytes and Wnt/beta-catenin signaling link nigrostriatal injury to repair in 1-methyl-4-phenyl-1,2,3,6-tetrahydropyridine model of Parkinson’s disease. *Neurobiol. Dis.* 41 508–527. 10.1016/j.nbd.2010.10.023 21056667 PMC3558878

[B27] L’Episcopo F. Tirolo C. Testa N. Caniglia S. Morale M. C. Impagnatiello F. (2011c). Switching the microglial harmful phenotype promotes lifelong restoration of subtantia nigra dopaminergic neurons from inflammatory neurodegeneration in aged mice. *Rejuvenation Res.* 14 411–424. 10.1089/rej.2010.1134 21793734

[B28] L’Episcopo F. Tirolo C. Caniglia S. Testa N. Morale M. C. Serapide M. F. (2014). Targeting Wnt signaling at the neuroimmune interface for dopaminergic neuroprotection/repair in Parkinson’s disease. *J. Mol. Cell Biol.* 6 13–26. 10.1093/jmcb/mjt053 24431301 PMC4061726

[B29] L’Episcopo F. Tirolo C. Testa N. Caniglia S. Morale M. C. Deleidi M. (2012). Plasticity of subventricular zone neuroprogenitors in MPTP (1-methyl-4-phenyl-1,2,3,6-tetrahydropyridine) mouse model of Parkinson’s disease involves cross talk between inflammatory and Wnt/beta-catenin signaling pathways: functional consequences for neuroprotection and repair. *J. Neurosci.* 32 2062–2085. 10.1523/JNEUROSCI.5259-11.2012 22323720 PMC3556384

[B30] L’Episcopo F. Tirolo C. Testa N. Caniglia S. Morale M. C. Impagnatiello F. (2013). Aging-induced Nrf2-ARE pathway disruption in the subventricular zone drives neurogenic impairment in parkinsonian mice via PI3K-Wnt/beta-catenin dysregulation. *J. Neurosci.* 33 1462–1485. 10.1523/JNEUROSCI.3206-12.2013 23345222 PMC3564519

[B31] Levin E. R. Gardner D. G. Samson W. K. (1998). Natriuretic peptides. *N. Engl. J. Med.* 339 321–328. 10.1056/NEJM199807303390507 9682046

[B32] Levites Y. Amit T. Mandel S. Youdim M. B. (2003). Neuroprotection and neurorescue against Abeta toxicity and PKC-dependent release of nonamyloidogenic soluble precursor protein by green tea polyphenol (-)-epigallocatechin-3-gallate. *FASEB J.* 17 952–954. 10.1096/fj.02-0881fje 12670874

[B33] Lopes F. M. da Motta L. L. De Bastiani M. A. Pfaffenseller B. Aguiar B. W. de Souza L. F. (2017). RA differentiation enhances dopaminergic features, changes redox parameters, and increases dopamine transporter dependency in 6-hydroxydopamine-induced neurotoxicity in SH-SY5Y cells. *Neurotox. Res.* 31 545–559. 10.1007/s12640-016-9699-0 28155214

[B34] Lopes F. M. Schröder R. da Frota M. L. Jr. Zanotto-Filho A. Müller C. B. Pires A. S. (2010). Comparison between proliferative and neuron-like SH-SY5Y cells as an in vitro model for Parkinson disease studies. *Brain Res.* 1337 85–94. 10.1016/j.brainres.2010.03.102 20380819

[B35] Mahinrad S. de Craen A. J. Yasar S. van Heemst D. Sabayan B. (2016). Natriuretic peptides in the central nervous system: novel targets for cognitive impairment. *Neurosci. Biobehav. Rev.* 68 148–156. 10.1016/j.neubiorev.2016.05.022 27229760

[B36] Martinat C. Shendelman S. Jonason A. Leete T. Beal M. F. Yang L. (2004). Sensitivity to oxidative stress in DJ-1-deficient dopamine neurons: an ES- derived cell model of primary Parkinsonism. *PLOS Biol.* 2:e327. 10.1371/journal.pbio.0020327 15502868 PMC521171

[B37] McKenzie J. C. Berman N. E. Thomas C. R. Young J. K. Compton L. Y. Cothran L. N. (1994). Atrial natriuretic peptide-like (ANP-LIR) and ANP prohormone immunoreactive astrocytes and neurons of human cerebral cortex. *Glia* 12 228–243. 10.1002/glia.440120308 7851990

[B38] Moon R. T. Kohn A. D. De Ferrari G. V. Kaykas A. (2004). WNT and beta-catenin signalling: diseases and therapies. *Nat. Rev. Genet.* 5 691–701. 10.1038/nrg1427 15372092

[B39] Morii N. Nakao K. Sugawara A. Sakamoto M. Suda M. Shimokura M. (1985). Occurrence of atrial natriuretic polypeptide in brain. *Biochem. Biophys. Res. Commun.* 127 413–419. 10.1016/S0006-291X(85)80176-5 3156596

[B40] Olanow C. W. Schapira A. H. (2013). Therapeutic prospects for Parkinson disease. *Ann. Neurol.* 74 337–347. 10.1002/ana.24011 24038341

[B41] Parish C. L. Arenas E. (2007). Stem-cell-based strategies for the treatment of Parkinson’s disease. *Neurodegener. Dis.* 4 339–347. 10.1159/000101892 17627139

[B42] Prado J. Baltrons M. A. Pifarre P. Garcia A. (2010). Glial cells as sources and targets of natriuretic peptides. *Neurochem. Int.* 57 367–374. 10.1016/j.neuint.2010.03.004 20302900

[B43] Prakash N. Brodski C. Naserke T. Puelles E. Gogoi R. Hall A. (2006). A Wnt1-regulated genetic network controls the identity and fate of midbrain-dopaminergic progenitors in vivo. *Development* 133 89–98. 10.1242/dev.02181 16339193

[B44] Prakash N. Wurst W. (2006). Genetic networks controlling the development of midbrain dopaminergic neurons. *J. Physiol.* 575 403–410. 10.1113/jphysiol.2006.113464 16825303 PMC1819467

[B45] Price T. O. Farr S. A. Niehoff M. L. Ercal N. Morley J. E. Shah G. N. (2015). Protective effect of topiramate on hyperglycemia-induced cerebral oxidative stress, pericyte loss and learning behavior in diabetic mice. *Int. Libr. Diabetes Metab.* 1 6–12. 26120599 PMC4479302

[B46] Quirion R. (1989). Receptor sites for atrial natriuretic factors in brain and associated structures: an overview. *Cell Mol. Neurobiol.* 9 45–55. 10.1007/BF00711442 2540911 PMC11567492

[B47] Robin N. C. Agoston Z. Biechele T. L. James R. G. Berndt J. D. Moon R. T. (2014). Simvastatin promotes adult hippocampal neurogenesis by enhancing Wnt/beta-catenin signaling. *Stem Cell Reports* 2 9–17. 10.1016/j.stemcr.2013.11.002 24511465 PMC3916759

[B48] Saito Y. (2010). Roles of atrial natriuretic peptide and its therapeutic use. *J. Cardiol.* 56 262–270. 10.1016/j.jjcc.2010.08.001 20884176

[B49] Sakurada K. Ohshima-Sakurada M. Palmer T. D. Gage F. H. (1999). Nurr1, an orphan nuclear receptor, is a transcriptional activator of endogenous tyrosine hydroxylase in neural progenitor cells derived from the adult brain. *Development* 126 4017–4026. 10457011 10.1242/dev.126.18.4017

[B50] Salinas P. C. (2012). Wnt signaling in the vertebrate central nervous system: from axon guidance to synaptic function. *Cold Spring Harb. Perspect. Biol.* 4:a008003. 10.1101/cshperspect.a008003 22300976 PMC3281574

[B51] Serafino A. Moroni N. Psaila R. Zonfrillo M. Andreola F. Wannenes F. (2012). Anti-proliferative effect of atrial natriuretic peptide on colorectal cancer cells: evidence for an Akt-mediated cross-talk between NHE-1 activity and Wnt/beta-catenin signaling. *Biochim. Biophys. Acta* 1822 1004–1018. 10.1016/j.bbadis.2012.02.016 22387884

[B52] Serafino A. Pierimarchi P. (2014). Atrial natriuretic peptide: a magic bullet for cancer therapy targeting Wnt signaling and cellular pH regulators. *Curr. Med. Chem.* 21 2401–2409. 10.2174/0929867321666140205140152 24524761 PMC4063317

[B53] Serafino A. Sferrazza G. Colini Baldeschi A. Nicotera G. Andreola F. Pittaluga E. (2017). Developing drugs that target the Wnt pathway: recent approaches in cancer and neurodegenerative diseases. *Expert Opin. Drug Discov.* 12 169–186. 10.1080/17460441.2017.1271321 27960558

[B54] Skelton W. P. T. Skelton M. Vesely D. L. (2013a). Cardiac hormones are potent inhibitors of secreted frizzled-related protein-3 in human cancer cells. *Exp. Ther. Med.* 5 475–478. 10.3892/etm.2012.806 23408665 PMC3570200

[B55] Skelton W. P. T. Skelton M. Vesely D. L. (2013b). Central role of beta-catenin in anticancer effects of cardiac hormones. *Anticancer Res.* 33 2409–2414. 23749889

[B56] Stambolic V. Woodgett J. R. (1994). Mitogen inactivation of glycogen synthase kinase-3 beta in intact cells via serine 9 phosphorylation. *Biochem. J.* 303 701–704. 10.1042/bj3030701 7980435 PMC1137602

[B57] Taira T. Saito Y. Niki T. Iguchi-Ariga S. M. Takahashi K. Ariga H. (2004). DJ-1 has a role in antioxidative stress to prevent cell death. *EMBO Rep.* 5 213–218. 10.1038/sj.embor.7400074 14749723 PMC1298985

[B58] Takekoshi K. Ishii K. Isobe K. Nomura F. Nammoku T. Nakai T. (2000). Effects of natriuretic peptides (ANP, BNP, CNP) on catecholamine synthesis and TH mRNA levels in PC12 cells. *Life Sci.* 66 L303–L311. 10.1016/S0024-3205(00)00549-X 10834306

[B59] Vesely D. L. (2009). Cardiac and renal hormones: anticancer effects in vitro and in vivo. *J. Investig. Med.* 57 22–28. 10.231/JIM.0b013e3181948b25 19092678

[B60] Vesely D. L. (2012). New anticancer agents: hormones made within the heart. *Anticancer Res.* 32 2515–2521. 22753708

[B61] Vesely D. L. (2013). Cardiac hormones for the treatment of cancer. *Endocr. Relat. Cancer* 20 R113–R125. 10.1530/ERC-13-0054 23533248

[B62] Wang R. Chen Z. Fu Y. Wei X. Liao J. Liu X. (2017). Plasma Cystatin C and high-density lipoprotein are important biomarkers of Alzheimer’s Disease and vascular dementia: a cross-sectional study. *Front. Aging Neurosci.* 9:26 10.3389/fnagi.2017.00026 PMC5294921 28223934

[B63] Wei L. Sun C. Lei M. Li G. Yi L. Luo F. (2013). Activation of Wnt/beta-catenin pathway by exogenous Wnt1 protects SH-SY5Y cells against 6-hydroxydopamine toxicity. *J. Mol. Neurosci.* 49 105–115. 10.1007/s12031-012-9900-8 23065334

[B64] Wei X. Gao H. Zou J. Liu X. Chen D. Liao J. (2016). Contra-directional Coupling of Nur77 and Nurr1 in neurodegeneration: a novel mechanism for memantine-induced anti-inflammation and anti-mitochondrial impairment. *Mol. Neurobiol.* 53 5876–5892. 10.1007/s12035-015-9477-7 26497037

[B65] Wilkins M. R. Redondo J. Brown L. A. (1997). The natriuretic-peptide family. *Lancet* 349 1307–1310. 10.1016/S0140-6736(96)07424-7 9142076

[B66] Wolozin B. Wang S. W. Li N. C. Lee A. Lee T. A. Kazis L. E. (2007). Simvastatin is associated with a reduced incidence of dementia and Parkinson’s disease. *BMC Med.* 5:20. 10.1186/1741-7015-5-20 17640385 PMC1955446

[B67] Xie H. R. Hu L. S. Li G. Y. (2010). SH-SY5Y human neuroblastoma cell line: in vitro cell model of dopaminergic neurons in Parkinson’s disease. *Chin. Med. J.* 123 1086–1092. 20497720

[B68] Xu Y. Yan J. Zhou P. Li J. Gao H. Xia Y. (2012). Neurotransmitter receptors and cognitive dysfunction in Alzheimer’s disease and Parkinson’s disease. *Prog. Neurobiol.* 97 1–13. 10.1016/j.pneurobio.2012.02.002 22387368 PMC3371373

[B69] Xu Y. Q. Long L. Yan J. Q. Wei L. Pan M. Q. Gao H. M. (2013). Simvastatin induces neuroprotection in 6-OHDA-lesioned PC12 via the PI3K/AKT/caspase 3 pathway and anti-inflammatory responses. *CNS Neurosci. Ther.* 19 170–177. 10.1111/cns.12053 23279934 PMC6493537

[B70] Yan W. Wu F. Morser J. Wu Q. (2000). Corin, a transmembrane cardiac serine protease, acts as a pro-atrial natriuretic peptide-converting enzyme. *Proc. Natl. Acad. Sci. U.S.A.* 97 8525–8529. 10.1073/pnas.150149097 10880574 PMC26981

[B71] Zhang L. Cen L. Qu S. Wei L. Mo M. Feng J. (2016). Enhancing Beta-catenin activity via GSK3beta inhibition protects PC12 cells against rotenone toxicity through Nurr1 induction. *PLOS ONE* 11:e0152931. 10.1371/journal.pone.0152931 27045591 PMC4821554

[B72] Zhang L. Yang X. Yang S. Zhang J. (2011). The Wnt /beta-catenin signaling pathway in the adult neurogenesis. *Eur. J. Neurosci.* 33 1–8. 10.1111/j.1460-9568.2010.7483.x 21073552

[B73] Zhou T. Zu G. Zhang X. Wang X. Li S. Gong X. (2016). Neuroprotective effects of ginsenoside Rg1 through the Wnt/beta-catenin signaling pathway in both in vivo and in vitro models of Parkinson’s disease. *Neuropharmacology* 101 480–489. 10.1016/j.neuropharm.2015.10.024 26525190

[B74] Zou J. Chen Z. Wei X. Chen Z. Fu Y. Yang X. (2017). Cystatin C as a potential therapeutic mediator against Parkinson’s disease via VEGF-induced angiogenesis and enhanced neuronal autophagy in neurovascular units. *Cell Death Dis.* 8:e2854. 10.1038/cddis.2017.240 28569795 PMC5520899

