# Supplementary material for: Atrial Natriuretic Peptide Acts as a Neuroprotective Agent in in Vitro Models of Parkinson’s Disease via Up-regulation of the Wnt/β-Catenin Pathway
Source: Front Aging Neurosci. 2018 Feb 1;10:20. doi: 10.3389/fnagi.2018.00020 (PMC5799264; doi:10.3389/fnagi.2018.00020)
Supplement: Supplementary file 1 [file Image_1.PDF]

“Atrial Natriuretic Peptide Acts As a Neuroprotective Agent in *In Vitro* Models of Parkinson's Disease via Up-Regulation of the Wnt/β-Catenin Pathway”.

Arianna Colini Baldeschi, Eugenia Pittaluga, Federica Andreola, Simona Rossi, Mauro Cozzolino, Giuseppe Nicotera, Gianluca Sferrazza, Pasquale Pierimarchi, Annalucia Serafino.

Correspondance: A. Serafino, Institute of Translational Pharmacology - National Research Council of Italy, Via Fosso del Cavaliere 100, 00133, Rome, Italy; Tel. : +39-06-45488202; Fax: +39-06-45488257 E-mail: annalucia.serafino@ift.cnr.it

SUPPLEMENTARY FIGURE 1

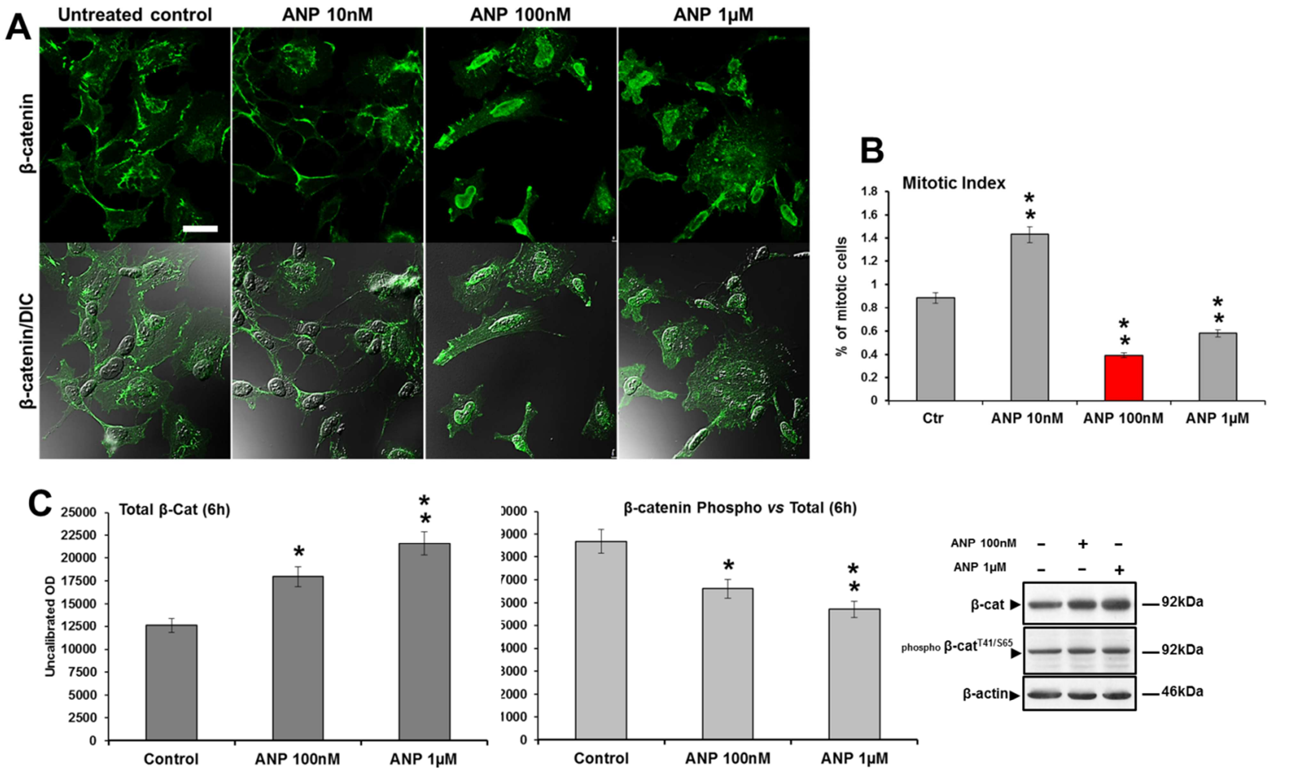

Supplementary Figure S1. Preliminary dose-response experiments performed on SHSY5Ywt cells for selecting the lowest effective and not toxic dose of ANP. Cells were treated with increasing concentrations of ANP (10 nM, 100 nM and 1 μM) and analyzed after 24 h for mitotic index (**B**), and intracellular localization of β-catenin by confocal microscopy (**A**), and after 6 h for the expression of total and phosphorylated β-catenin by WB (**C**). For confocal microscopy, cell morphology was visualized by differential interference contrast (DIC) and both β-catenin single staining and merged images with DIC are shown. Bar in A): 25 μm. ANP 100 nM has been selected as the lowest dose inducing nuclear β-catenin translocation (A), cell proliferation arrest (**B**), increased levels of total β-catenin and a concomitant decrease on β-catenin phosphorylation at T41/S45 (decreased β-catenin degradation) (**C**). Significance *vs* untreated control (Student’s t test and a p value threshold of < 0.05): *, $p < 0.05$; **, $p < 0.01$; $n = 3$.
