# Supplementary material for: Atrial Natriuretic Peptide Acts as a Neuroprotective Agent in in Vitro Models of Parkinson’s Disease via Up-regulation of the Wnt/β-Catenin Pathway
Source: Front Aging Neurosci. 2018 Feb 1;10:20. doi: 10.3389/fnagi.2018.00020 (PMC5799264; doi:10.3389/fnagi.2018.00020)
Supplement: Supplementary file 2 [file Image_2.PDF]

"Atrial Natriuretic Peptide Acts As a Neuroprotective Agent in *In Vitro* Models of Parkinson's Disease via Up-Regulation of the Wnt/β-Catenin Pathway".

Arianna Colini Baldeschi, Eugenia Pittaluga, Federica Andreola, Simona Rossi, Mauro Cozzolino, Giuseppe Nicotera, Gianluca Sferrazza, Pasquale Pierimarchi, Annalucia Serafino.

Correspondance: A. Serafino, Institute of Translational Pharmacology - National Research Council of Italy, Via Fosso del Cavaliere 100, 00133, Rome, Italy; Tel. : +39-06-45488202; Fax: +39-06-45488257
E-mail: annalucia.serafino@ift.cnr.it

SUPPLEMENTARY FIGURE 2

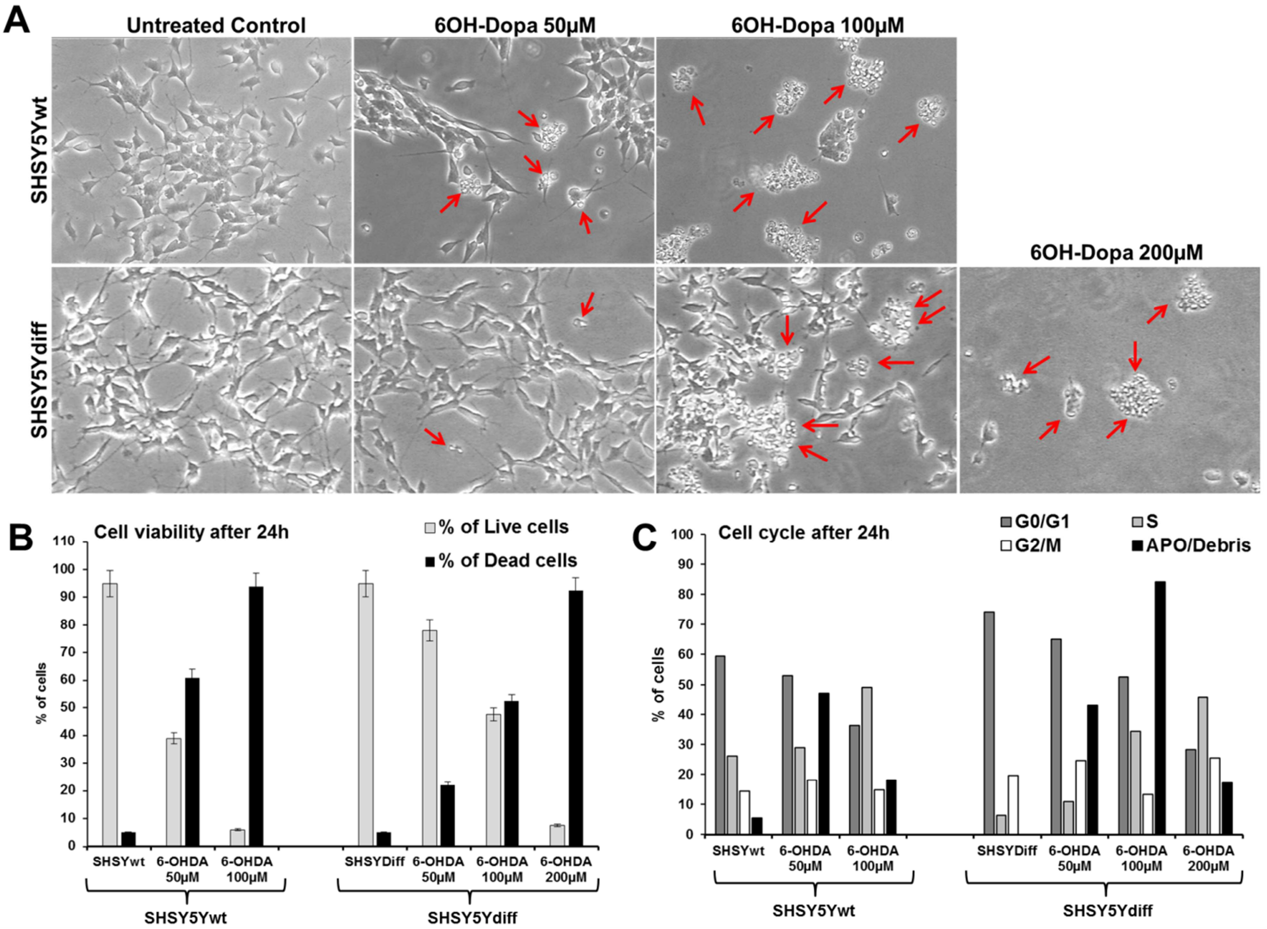

Supplementary Figure S2. Dose response experiments of 6-OHDA toxicity on SHSY5Ywt and SHSY5Ydiff cells. Proliferative SHSY5Ywt and RA-differentiated SHSY5Ydiff cells were exposed to increasing concentration of 6-OHDA (25, 50, 100, 200 and 400 µM) for 24 h and cytotoxicity was assessed by evaluating cell survival by optical microscopy (**A**), cell viability assay (**B**) and cell cycle analysis (**C**). In a first screening, the concentration of 25, and 400 µM resulted ineffective or too aggressive, respectively, and were excluded from the tests in triplicate. **A**) Phase contrast microscopy of SHSY5Ywt (*upper panels*) and SHSY5Ydiff (*lower panels*) cells treated 50, 100 and 200µM 6-OHDA; red arrows point to dying/dead cells detached from the adhering monolayer. Original magnification: 20x. **B**) Cell viability assay performed after 24 h of 6-OHDA treatment by Trypan blue dye exclusion method; results are reported as percentage of live/dead cells ± SD, are mean of three independent experiments ($n = 3$). **C)** Cytofluorimetric analysis of DNA content in SHSY5Ywt and SHSY5Ydiff cells, performed after 24 h of 6-OHDA treatment.
